# Biomarkers and genotypes in patients with Central nervous system infection caused by enterovirus

**DOI:** 10.1080/23744235.2024.2345712

**Published:** 2024-05-17

**Authors:** Karolina Alsén, Marianela Patzi Churqui, Helene Norder, Karolina Rembeck, Henrik Zetterberg, Kaj Blennow, Fredrika Sahlgren, Anna Grahn

**Affiliations:** a Institute of Biomedicine, Department of Infectious Diseases, University of Gothenburg, Gothenburg, Sweden; b Department of Infectious diseases, Västra Götaland Region, Sahlgrenska University Hospital, Gothenburg, Sweden; c Department of Clinical Microbiology, Region Västra Götaland, Sahlgrenska University Hospital, Gothenburg, Sweden; d Inst. of Neuroscience and Physiology, University of Gothenburg, Mölndal, Sweden; e Clinical Neurochemistry Lab, Sahlgrenska University Hospital, Mölndal, Sweden; f Department of Neurodegenerative Disease, UCL Institute of Neurology, Queen Square, London, UK; g UK Dementia Research Institute at UCL, London, UK; h Hong Kong Center for Neurodegenerative Diseases, Hong Kong, China; i Wisconsin Alzheimer’s Disease Research Center, University of Wisconsin School of Medicine and Public Health, University of Wisconsin-Madison, Madison, Wisconsin, USA; j Institut du Cerveau et de la Moelle épinière (ICM), Pitié-Salpêtrière Hospital, Sorbonne Université, Paris, France; k University of Science and Technology of China, Hefei, P.R. China; l Department of Infectious Diseases, Skövde Hospital, Skövde, Sweden

**Keywords:** Enterovirus, central nervous system infection, biomarkers, genotypes, meningitis

## Abstract

**Purpose:**

Enteroviruses (EV) comprises many different types and are the most common cause of aseptic meningitis. How the virus affects the brain including potential differences between types are largely unknown. Measuring biomarkers in CSF is a tool to estimate brain damage caused by CNS infections.

**Methods:**

A retrospective study was performed in samples from 38 patients with acute neurological manifestations and positive CSF-EV RNA (*n* = 37) or serum-IgM (*n* = 1). The EV in 17 samples were typed by sequencing. The biomarkers neurofilament light (NFL), glial fibrillary acidic protein (GFAP), S-100B protein, amyloid-β (Aβ) 40 and Aβ42, total-tau (T-tau) and phosphorylated tau (P-tau) were measured and compared with data derived from a control group (*n* = 19).

**Results:**

There were no increased levels of GFAP (*p* ≤ 0.1) nor NFL (*p* ≤ 0.1) in the CSF of patients with EV meningitis (*n* = 38) compared with controls. However, we found decreased levels of Aβ42 (*p* < 0.001), Aβ40 (*p* < 0.001), T-tau (*p* ≥ 0.01), P-tau (*p* ≤ 0.001) and S-100B (*p* ≤ 0.001). E30 (*n* = 9) and CVB5 (*n* = 6) were the most frequent EV-types identified, but no differences in biomarker levels or other clinical parameters were found between the infecting virus type. Seven patients who were followed for longer than one month reported remaining cognitive impairment, although no correlations with biomarker concentrations were observed.

**Conclusion:**

There are no indication of neuronal or astrocyte damage in patients with EV meningitis. Yet, decreased concentrations of Aβ40, Aβ42, P-tau and T-tau were shown, a finding of unknown importance. Cognitive impairment after acute disease occurs, but with only a limited number of patients analysed, no conclusion can be drawn concerning any association with biomarker levels or EV types.

## Introduction

Enterovirus (EV) may cause a wide spectrum of diseases including mild gastrointestinal and respiratory infections, hand-foot-and-mouth disease, herpangina and more severe diseases such as myopericarditis, pancreatitis, hepatitis, meningitis, encephalitis with paralysis and flaccid myelitis [1]. Children and young adults are more frequently affected. EV is the most common virus found in cerebrospinal fluid (CSF) in patients with aseptic meningitis [2,3]. EV meningitis is generally considered benign. A Danish study found no impact of long-term survival, health or social nor educational functioning [1]. However, in a British study, quality of life was reported to be clearly reduced in the EV meningitis group compared with the UK population at follow-up, up to 48 weeks after acute admission [2].

The viruses in the genus Enterovirus in the *Picornaviridae* family are small RNA viruses. They form 15 species, Enterovirus A-J and Rhinovirus A-C. In species EV-A–EV-D there are 116 different virus types, 108 of these infect humans. These viruses are a significant cause of morbidity and mortality worldwide [4]. Some types are more neurotropic than others; polioviruses, known to cause severe neurological disability, belong to EV-C. EV-A71 is frequently causing severe outbreaks with high mortality among young children in East Asia [4,5]. Multiple different types are circulating around the world, sometimes causing outbreaks. There were worldwide outbreaks of enterovirus D68 (EV-D68) between 2014 and 2018. The virus, previously considered a harmless virus causing common cold, had changed genetically, and caused serious respiratory and neurological disease during the outbreaks [6,7].

Though EV are the most common virus causing aseptic meningitis, there is a lack of knowledge in how the virus affects the brain including potential differences between different types, both in short and long-term perspective. The results in recent studies differ according to the long-term outcome [1,2]. Measuring biomarkers in CSF is a tool to estimate potential brain damage caused by CNS infections and may also be helpful to foresee the prognosis [8–10].

The light subunit of neurofilament protein (NFL), glial fibrillary acidic protein (GFAP), S-100B protein, beta-amyloid (Aβ40) and (Aβ42), total-tau (T-tau) and phosphorylated tau (P-tau) are all biomarkers used to assess brain damage and/or neurodegeneration. NFL is a biomarker expressed by the neurons and indicates neuroaxonal injury [11]. Increased NFL levels in CSF have been measured in patients with CNS infection caused by varicella-zoster virus (VZV) and herpes simplex virus (HSV) [12,13]. S-100B and GFAP both reflect astroglial damage and/or activation. In the CSF of patients with VZV encephalitis, GFAP has been shown to be increased, whilst S-100B was decreased compared with controls [13]. T-tau and P-tau reflects cortical axonal degeneration and neurofibrillary tangle pathology, while Aβ42 reflects amyloid plaque pathology, which are hallmarks of Alzheimer’s disease [14,15]. In children with CNS infections including EV infection, studies on biomarker levels in CSF have shown inconclusive results. No changes in beta-amyloid concentrations were reported in one study, in contrast to another, where CSF-Aβ42 was significantly decreased in 42 children diagnosed with EV meningitis [15,16].

In this study, our aim was to investigate the concentrations of CSF biomarkers indicating CNS injury in patients with EV CNS infection. Secondly, we wanted to study the different EV types causing CNS infection in relation to biomarker concentrations and to clinical parameters.

## Material and methods

### Patients and controls

The inclusion criteria of the study were patients ≥18 years, with acute EV CNS infection admitted to one of the clinics of infectious diseases at Norra Älvsborgs Länssjukhus (NÄL), Skövde Hospital (SkaS) or Sahlgrenska University Hospital, in the county of Western Gotaland, Sweden, between 2008 and 2018. Detection of EV RNA by PCR in CSF, nasal mucus or faeces, or detection of EV IgM antibodies in serum, were used for microbiological verification. By international classification of diseases (ICD) codes, patients diagnosed with EV CNS infection were identified. The ICD codes A87.0, A87.9, G03.9, G04.9, A86.9, and A85.0 were used. Exclusion criteria were absence of microbiological verification of the EV infection. Through personal identification numbers, the CSF samples stored at the Virological Laboratory of Sahlgrenska University Hospital were located. Patients with enough CSF for both genotyping and biomarker analyses were finally included. Clinical information was obtained through medical records and data including gender, age, neurological symptoms and sequelae, duration of admission, cell count and date of diagnosis were registered.

All patients except one were diagnosed by positive PCR for EV in CSF. The patient with a negative PCR in CSF were instead diagnosed by a positive serology with detection of EV IgM in serum in conjunction with symptoms of viral meningitis and absence of PCR positivity in CSF of other possible pathogens.

Nineteen non-infectious subjects were selected as a control group. The controls were selected for age and gender to match the patients in the study group. All controls had done a lumbar puncture at the Clinic of Infectious Diseases at Sahlgrenska University hospital as a part of investigestion of suspected CNS infection. The controls had normal CSF lymphocyte counts, albumin concentrations and a normal neurological examination. In addition, no CNS disease was diagnosed within one year after sampling, according to medical records.

### Management of CSF samples

Non-centrifuged CSF were analysed for cell counts, and for EV RNA by either a multiplex PCR [17] and/or a quantitative in-house PCR [18] at the time for acute disease. All samples diagnosed by the multiplex PCR at the first stage, introduced in the routine diagnostics in April 2017, were reanalysed by the quantitative PCR for verification. Sensitivity of the multiplex PCR used in this study is reported to 89% for EV [17]. Patient CSF samples were stored non-centrifuged at −70 °C or −20 °C. CSF samples from control subjects were stored at −70 °C as part of a biobank. These stored samples were then used for further analyses.

For analysis of biomarkers of neurodamage, CSF were sent to the neurochemistry laboratory at Sahlgrenska University Hospital. All CSF samples from the patients and the control subjects were analysed on one occasion using one batch of reagents for the biomarkers Aβ42, Aβ40, S-100B, P-tau (phosphorylated at amino acid 181), T-tau, GFAP and NFL. Aβ42, Aβ40, P-tau, and T-tau concentrations were measured by Lumipulse (Fujirebio, Ghent, Belgium), as previously described [19]. S-100B concentration was measured using the Elecsys® S100 assay on a cobas e601 instrument (Roche Diagnostics, Penzberg, Germany). GFAP and NFL concentrations were measured using in-house enzyme-linked immunosorbent assays (ELISAs) [11,20].

### Cell culture and sequencing of EV strains

To improve the EV characterisation, cell culture for isolation of EV was performed using suspensions of tree cell lines: Green monkey kidney cells (GMK), colorectal adenocarcinoma (Caco-2), and human lung embryonal fibroblast (MRC-5). For viral infection/isolation, CSF and NPH samples were filtered using a 0.45 um membrane, and then applied to the cell suspensions that were seeded in 96-well plates. For faeces samples, approximately 0.1 g of the sample was mixed with 500 μl of complete medium, followed by centrifugation, filtration and infection as previously described. The viruses in the samples were isolated as previously described [21]. Supernatants from each well of the cultured samples and positive isolates were collected for further sequencing.

Amplification and sequencing were performed of conserved and variable genomic regions of the viral encoded structural protein VP1 and the 5’untranlsated region 5’UTR. Briefly, total nucleic acid were extracted from 200 µL of the positive sample or isolated supernatant using the DNeasy Blood and Tissue kit, (Qiagen, Hilden, Germany) according to the manufacturer’s instructions. Before amplifying the different regions, cDNA was first made using random primers and the High-capacity cDNA Reverse Transcription kit with RNase inhibitor (Applied Biosystems).

The PCR systems for amplification of the regions were performed on (Applied Biosystems) instruments and all samples were tested in duplicates as previously described [21,22]. The 5’UTR region was first amplified using the primers EVP4AS and EV140S, followed by a semi-nested PCR using EV140S and EV170AS. For the VP1 region, a combination of primers ENT224S and ENT222AS was used, and a subsequent semi-nested PCR using AS89S and AN88AS primers. The primer sequences and combinations for the PCR and nested PCR are listed in the Supplementary Table 1. Amplicons were extracted/purified using sense and antisense primers in the PCRs and the BigDye Terminator 3.1 (Applied Biosystems) cycle sequencing kit by Sanger sequencing using the Mix2seq service (Eurofins genomics; Ebersberg, Germany). All sequences were analysed using the SeqMan Ultra program in the DNAstar Lasergene package version 17.0 (DNASTAR, Inc; Madison, USA).

Phylogenetic analyses of the sequences obtained were performed by UPGMA and neighor joining methods in the MEGA-X program package version 10.0.5 [23] to classify the analysed viruses into different types by using corresponding sequences available for all reference EV types.

### Management of NPH samples, faeces samples and serum samples

In some patients with suspected EV CNS disease, further analyses were performed at the time for acute disease for detection of EV RNA in nasopharyngeal swaps (*n* = 3) or faeces (*n* = 2) by a multiplex and an in-house PCR [17,21]. For one patient E30 could be identified in both CSF and faeces. In one patient with negative EV PCR in CSF, further analyses in serum were performed for detection of EV IgM with an in-house ELISA.

### Statistical analysis

Statistical analysis was performed using GraphPad PRISM 9.0 (GraphPad Software, San Diego, USA). Non-parametric statistics as Mann–Whitney *U* test for group comparisons were used and descriptive statistics were used for IQR and range. A *p*-value of < 0.05 was considered significant.

### Ethics

The National Swedish Ethical Review Authority approved the study (Dnr 2019-02927).

## Results

### Patients

Thirty-eight patients (21 male, 17 female) with a median age of 28 years (range 17–57) with enterovirus CNS infection and sufficient CSF for biomarker analyses and viral genotyping were included in the study. All patients were diagnosed with meningitis with symptoms including headache, fever, nausea and/or photophobia. None of them had encephalitis nor myelitis. A lumbar puncture was performed within 24 h (range 1–5 days) after onset of symptoms of CNS infection. A CT scan of the brain was performed in 15 patients, all with normal outcome. No MRI was done. Three patients had previously been diagnosed with meningitis, two of unknown viral cause and one due to VZV. One patient had a history of cerebral haemorrhage and one suffered from mild mental retardation and ADHD. No other comorbidity was reported nor was any patient immunocompromised. Baseline characteristics for patients and controls are presented in Table 1.

**Table 1.** Baseline characteristics, CSF findings and genotype of 38 patients with enterovirus CNS infection and their controls (*n* = 19).

	E30 (*n* = 9)	CVB5 (*n* = 6)	E6 (*n* = 2)	Not genotyped (*n* = 21)	Total	Control group (*n* = 20)
Sex M/F	5/4	3/3	2/0	11/10	21/17	10/9
Age (years)	27 (20–41)	29 (17–37)	32 (31–33)	31 (19–57)	28.5 (17–57)	36 (21–48)
Duration of admission (days)	2 (2–5)	1 (1–2)	1 (1–1)	1 (1–6)	1.5 (1–6)	NA
CSF leukocyte count (10^6^/L) (*n* = 37)	240 (27–656)	202 (50–1191)	132 (66–197)	82 (12–334)	127 (12–1191)	<3
CSF mononuclear cell count (10^6^/L) (*n* = 37)	111(18–576)	64 (38–1134)	105 (57–152)	74 (6–248)	79 (6–1134)	<3
CSF polymorphonuclear cell count (10^6^/L) (*n* = 37)	59 (0–192)	50 (5–334)	27 (9–45)	8.5 (3–130)	14 (3–334)	<3
CSF albumin (mg/L) (*n* = 32)	543 (226–877)	360 (132–720)	414 (341–487)	402 (127–819)	463 (127–877)	Within reference value.
Enterovirus RNA in CSF by PCR (10-log Geq/ml) (*n* = 37)	4.4 (3.4–5.1)	2.6 (1.7–4.9)	4.0 (3.4–4.5)	3.7 (1.7–4.9)	3.9 (1.7–5.1)	NA

### Enterovirus genotypes – patients and cell counts

Of the 38 available CSF samples, 17 could be EV-genotyped. E30 (*n* = 9) and CVB5 (*n* = 6) were most frequently found, and two were genotyped as E6. One in the E30 group had previously had a meningitis and one had a mild mental retardation and ADHD. The remaining 7 patients were healthy. In the CVB5 group, one had a history of intracerebral haemorrhage, no other co-morbidity was noted. The patients were admitted to the hospital for 1–2 days (median 1.5 days, range 1–6, *n* = 38). Patients with genotype E30 (*n* = 9) were in general admitted 1 day longer than patients with EV CVB5 (*n* = 6) (*p* = 0.0012). There were no differences in time of CSF sampling after onset of symptoms between the different genotypes.

There were no differences when comparing CSF cell counts in patients with genotype E30 and CVB5. CSF leucocyte counts in patients with E30 was median 240 × 10^6^/L (range 27-656 × 10^6^/L) and in the CVB5 group, 202 × 10^6^/L(50-1191 x10^6^/L) (p = ns). Both groups had CSF monocytocis, with median 111 × 10^6^ monocytes/L (range 18-576) in genotype E30 and 64 × 10^6^ monocytes/L (68-1130) in patients with CVB5 genotype (p = ns). The CSF polymorphonuclear cell count in the E30 group was in median 59 × 10^6^/L 0–192 × 10^6^/L) compared with 50 × 10^6^/L (5-334 x10^6^/L) in the CVB5 group (p = ns). The CSF cell counts of patients with genotype E6 were not included in the analyses because they were few (*n* = 2).

### Neurological sequelae

Most patients (*n* = 30) had a short-term follow-up within four weeks after discharge. Eight patients had no follow up. Of the 30 patients, seven patients still suffered from neurological sequelae at follow-up more than one month after discharge, see Table 2. The symptoms of these seven patients consisted of brain fatigue, memory difficulties, headache, concentration difficulties, sensitivity to sounds and vertigo. Several of the patients received further help from neurorehabilitation. Viruses in samples from only 2 of the 7 patients could be typed and were verified to be E30.

**Table 2.** Neurological sequelae at follow-up in 38 patients with CNS infection caused by enterovirus.

Genotype of enterovirus	No. of patients with sequelae at follow up 0–2 weeks after discharge^a^	No. of patients with sequelae at follow up 2–4 weeks after discharge^a^	No. of patients with sequelae >4 weeks after discharge.	No. of patients without follow-up
E 30 (*n* = 9)	6/8	3/4	2/2	1
CVB5 (*n* = 6)	3/5	0/1	0	1
E6 (*n* = 2)	2/2	0	0	0
Not genotyped (*n* = 21)	12/15	5/7	5/5	6
Total (*n* = 38)	23/30	8/12	7/7	8

^a^ Carry backward was used, meaning that patients that reported sequelae at one follow-up occasion were also assumed to have sequelae at previous follow-ups.

### Seasonal variation

**Figure 1.**
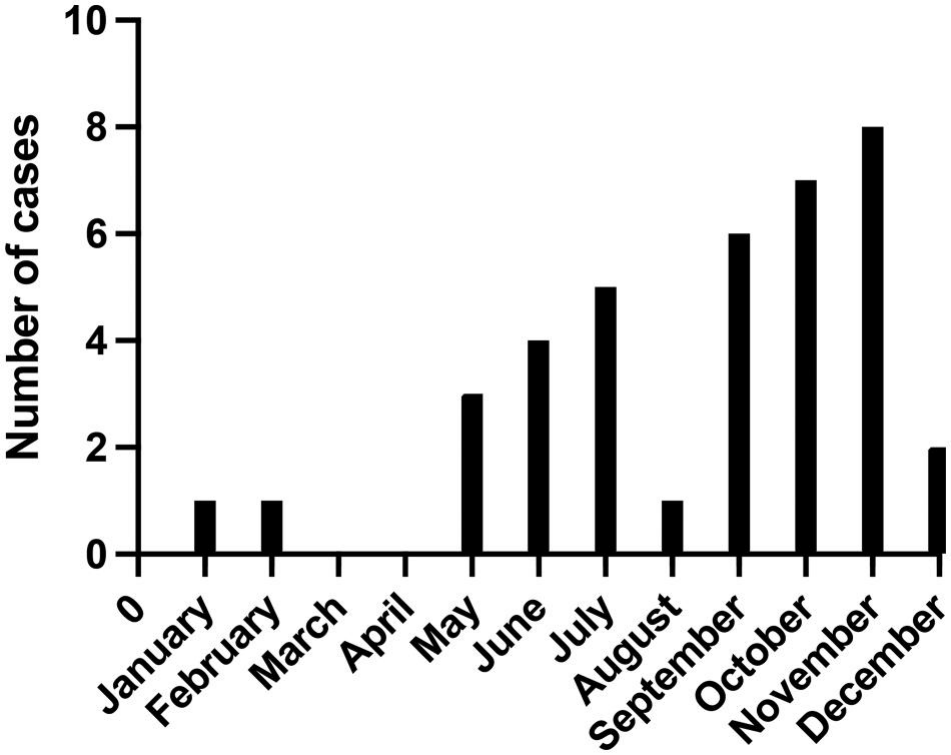
Number of patients with EV CNS infection per month during the study period of 2008 until 2018 (total number of patients: 38).

Most patients (*n* = 22) developed symptoms of meningitis between June and November, see Figure 1. One was diagnosed in January and one in February and two patients were diagnosed in May and December, respectively. There was no seasonal variation comparing the patients with genotype E30 to the patients with genotype CVB5.

### CSF biomarkers

**Figure 2.**
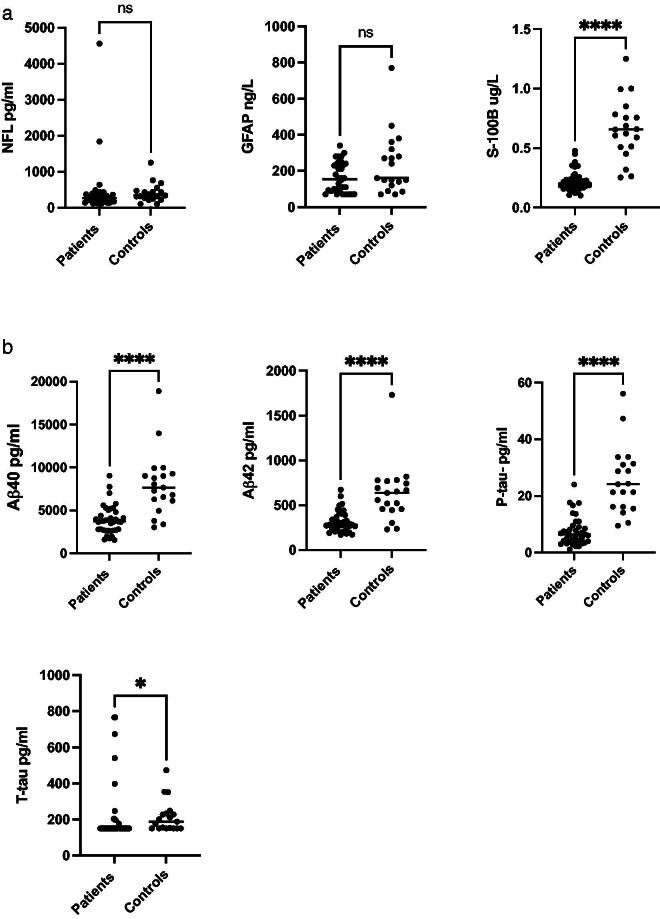
(a) NFL, GFAP and S-100B levels in CSF of patients with EV CNS infection (*n* = 38) and controls (*n* = 19). (b) Aβ40, Aβ42, P-tau and T-tau levels in CSF of patients with EV CNS infection (*n* = 38) and controls (*n* = 19).

Among the biomarkers analysed in patients with EV CNS infection (*n* = 38), the CSF concentrations of Aβ42, Aβ40, S-100B and P-tau, were all significantly decreased compared with the control group, see Figure 2a, b.

CSF Aβ42 concentration (*n* = 38) was lower compared with the controls. In the patient group the level was 283 pg/mL (median); IQR 234–395 compared with 637 pg/mL; 456–769 (*p* ≤ 0.0001) in the control group. There were also lower concentrations of Aβ40 (*n* = 38), in the patient group; 3740 pg/mL; IQR 2680-4860 compared with 7660 pg/mL; 6120-9280 in the control group (*p* ≤ 0.0001). The CSF Aβ42/Aβ40 ratio in the patient group was 0.078 pg/mL; IQR 0.073–0.099 compared with 0.082 pg/mL; 0.075–0.087 in the control group (*p* = 0.8175). Median CSF concentration of S-100B in the patient group (*n* = 38) was 0.197 ug/L; IQR 0.16–0.56 compared with the control group, where median was 0.656 ug/L; 0.51–0.78 (*p* ≤ 0.0001). The levels of P-tau (*n* = 38) were decreased in the patients compared with controls; 6.4 pg/mL; IQR 3.95–9.23 versus 24.1 pg/mL; 16.2–31.4 (*p* ≤ 0.0001), The CSF concentrations of T-tau were also decreased in the patients; 150 pg/mL; IQR 150–182 compared with the controls, 188 pg/mL; 150–233 (*p* ≤ 0.0143).

There were no significant differences comparing CSF concentrations of GFAP and NFL in patients with EV CNS infections compared with controls. GFAP (*n* = 36) concentrations were median 155 ng/L; IQR 94–238 in the patient group compared with 160 ng/L; 120–320 in the control group (*p* = 0.134). Levels of CSF NFL (*n* = 37) were 260 pg/mL; IQR 170–380 in the patient group versus 340 pg/mL; 250–470 in the controls (*p* = 0.098).

No differences in CSF biomarker concentrations were shown comparing patients with EV CNS infections caused by E30 to the ones caused by CVB5.

When comparing the levels of CSF biomarkers in patients that were fully recovered (*n* = 7) with patients with remaining sequelae (*n* = 7) at the follow up 4 weeks after discharge, there were no significant differences of any of the biomarkers. Data not shown.

## Discussion

Enterovirus are the most common cause of aseptic meningitis. In this retrospective study on biomarkers in patients with EV CNS infection, we found significantly lower CSF concentrations of Aβ42, Aβ40, T-tau, P-tau and S-100B, and normal levels of NFL and GFAP in patients with EV meningitis compared with controls.

Neuroinflammation is common for patients with CNS infections and other neurological diseases and seems able to affect both CSF amyloid and tau metabolism. Increased levels of CSF T-tau and P-tau reflect damage to or degenerative changes in neurons and, in CNS infections, an increase of mainly T-tau has been interpreted as neuronal injury caused by the infection and/or neuroinflammation [24]. The decreased levels of T-tau and P-tau seen in our study are difficult to explain but could potentially reflect decreased neuronal activity-dependent secretion of these proteins [25]. In a mass-spectrometric assay increased concentrations af phosphatidylcholines, which are important constituents of the cell membranes in eukaryotes, were found in patients with EV meningitis [26]. The authors proposed specific pathological processes within the meninges and that the finding may reflect subtle forms of meningeal cell membrane damage by the virus, which could possibly lead to a reduction of tau secretion. Another possibility could be that all patients included in our study were diagnosed with meningitis which is confined to the subarachnoid space and meninges with less involvement of the neurons in other parts of the brain. However, another study including patients with EV meningitis did show elevated levels of P-tau [27].

The decrease of Aβ40 and Aβ42 in the CSF of the patients with EV meningitis in our study is in line with the results from other studies including children with EV meningitis [16] and in patients with other CNS infections such as HSV-1 encephalitis, CNS opportunistic infections and patients with acute bacterial meningitis [28]. In addition, we found no changes in the Aβ42/Aβ40 ratio when comparing patients with controls. In Alzheimer’s disease, there is a selective reduction in CSF Aβ42 concentration (because of sequestration of this longer form of Aβ in amyloid plaques in the brain parenchyma), whilst Aβ40 stays normal [29]. In neuroinfectious and neuroinflammatory conditions, however, both Aβ forms decrease, along with secreted forms of amyloid precursor protein (APP). The mechanisms behind this are not known but may reflect altered APP expression and/or processing and release from neurons affected by neuroinflammation [30].

Moreover, we found significantly decreased CSF levels of S-100B compared with the control group. In VZV CNS infections, S-100B has been reported to be decreased in the CSF [13] while normal CSF levels of S-100B has been observed in other CNS infections such as neuroborreliosis [31] and in tick-borne encephalitis [32]. Decreased or normal levels of S100B in conjunction with normal GFAP indicates no damage of the astrocyte cell membranes.

NFL and GFAP were also analysed, but we found no significant differences in CSF concentrations between the patient group and the control group, which indicates no or only minor damage of the neurons and astrocytes caused by EV meningitis.

E30 and CVB5 were the most frequent types infecting the patients in our study, which is in line with previous findings. When The Public Health Authority in Sweden did a compilation in 2020 of the enterovirus serotypes causing CNS infection, they found that CVB5 and E30 were among the five most common types found and in two separate studies from Greece and Denmark 2021; E30, CVB5 and E6 accounted for the majority of all types [33,34]. Although enteroviruses are dynamic viruses these findings suggest that these virus types are those that have mainly circulated in Europe in recent years. We found that patients infected by E30 in general were admitted for one day longer, compared with those infected with CVB5, but there were no differences in brain biomarker concentrations or other indications of differences in severity between these two virus types. However, the groups were small, 9 respective 6 patients, which makes it precarious to draw any conclusions.

It should also be mentioned that in recent years human parechovirus (HPeV) has been identified as a virus closely related to EV presenting with similar symptoms in infants including meningoencephalitis and sepsis-like illness [35,36]. However, HPeV seems primarily to infect infants and is not as common as EV as a cause of viral meningitis in adults. Both HPeV and EV belongs to the family of *Picornaviridae* but they require individual RT-PCR assays for detection [36]. HPeVas well as EV are included in the multiplex-assay that was used for a limited number of CSF samples in this study to detect EV RNA [18]. Yet, in this study including patients ≥ 18 years of age we choose not to include HPeVand to only focus on EV.

Remaining physical or cognitive deficits seems rare in patients with EV CNS infections [34] but results in recent studies differ according to the long-term outcome [1,2]. We found that 7 out of 38 patients had remaining neurological sequelae more than 4 weeks after discharge. In the remaining 23 patients, follow up terminated within 4 weeks but several of these patients suffered from remaining neurological symptoms. Since the follow-up of these 23 patients were ended from the infectious disease clinic after 4 weeks, it is unclear whether these patients recovered or sought another healthcare institution. Long-term follow-up studies of patients with EV CNS infections would be of value.

Even though EV infections tend to infect children this study focused on adults and the median age (28.5) of the patients in our study supports that EV CNS infection mainly affect young adults [33,34]. EV infections are reported to be most common in summer and autumn [33] which was also reflected in our study where the majority of the patients fell ill in the fall.

Clear limitations of the study are that only few patients were followed up and that only 17 of the included 38 patients with EV CNS infection could be genotyped. We found no correlations of biomarker concentrations to neurological sequelae or EV types, but this should be further investigated in a larger study cohort.

## Conclusion

We found no increased levels of GFAP and NFL in the CSF of patients with EV meningitis, indicating no or only minor damage on neurons and astrocytes. Despite this, several patients reported remaining cognitive impairment lasting over one month. The finding of decreased levels of Aβ42, Aβ40, T-tau, P-tau and S-100B is difficult to explain and needs to be confirmed. In this small study, E30 and CVB5 were the most common EV types, and the clinical courses they caused had no significant differences.

## Supplementary Material

Supplemental Material

Supplemental Material

## Data Availability

The datasets generated during and/or analysed during the current study are not publicly available but are available from the corresponding author on reasonable request.

## References

[1] Omland LH, Holm-Hansen C, Lebech A-M, et al. Long-term survival, health, social functioning, and education in patients with an enterovirus Central nervous system infection, Denmark, 1997-2016. J Infect Dis. 2020;222(4):619–627. doi: 10.1093/infdis/jiaa151. 32236420

[2] McGill F, Griffiths MJ, Bonnett LJ, et al. Incidence, aetiology, and sequelae of viral meningitis in UK adults: a multicentre prospective observational cohort study. Lancet Infect Dis. 2018;18(9):992–1003. doi: 10.1016/S1473-3099(18)30245-7. 30153934 PMC6105576

[3] de Crom SC, Rossen JW, van Furth AM, et al. Enterovirus and parechovirus infection in children: a brief overview. Eur J Pediatr. 2016;175(8):1023–1029. doi: 10.1007/s00431-016-2725-7. 27156106 PMC4930465

[4] B'Krong NTTC, Minh NNQ, Qui PT, et al. Enterovirus serotypes in patients with Central nervous system and respiratory infections in Viet Nam 1997-2010. Virol J. 2018;15(1):69. doi: 10.1186/s12985-018-0980-0. 29650033 PMC5897964

[5] Taravilla CN, Pérez-Sebastián I, Salido AG, et al. Enterovirus A71 infection and neurologic disease, Madrid, Spain, 2016. Emerg Infect Dis. 2019;25(1):25–32. doi: 10.3201/eid2501.181089. 30560775 PMC6302576

[6] Holm-Hansen CC, Midgley SE, Fischer TK. Global emergence of enterovirus D68: a systematic review. Lancet Infect Dis. 2016;16(5):e64–e75. doi: 10.1016/S1473-3099(15)00543-5. 26929196

[7] Messacar K, Abzug MJ, Dominguez SR. 2014 Outbreak of enterovirus D68 in North america. J Med Virol. 2016;88(5):739–745. doi: 10.1002/jmv.24410. 26489019

[8] Petzold A. CSF biomarkers for improved prognostic accuracy in acute CNS disease. Neurol Res. 2007;29(7):691–708. doi: 10.1179/016164107X240080. 18173909

[9] Chekrouni N, van Soest TM, Brouwer MC, et al. CSF neurofilament light chain concentrations predict outcome in bacterial meningitis. Neurol Neuroimmunol Neuroinflamm. 2022;9(1):e1123. doi: 10.1212/NXI.0000000000001123. PMC8669658 34903639

[10] Tyrberg T, Nilsson S, Blennow K, et al. Serum and cerebrospinal fluid neurofilament light chain in patients with Central nervous system infections caused by varicella-zoster virus. J Neurovirol. 2020;26(5):719–726. doi: 10.1007/s13365-020-00889-2. 32816287 PMC7532135

[11] Gaetani L, Hoglund K, Parnetti L, et al. A new enzyme-linked immunosorbent assay for neurofilament light in cerebrospinal fluid: analytical validation and clinical evaluation. Alzheimers Res Ther. 2018;10(1):8. 29370869 10.1186/s13195-018-0339-1 PMC6389166

[12] Westman G, Aurelius E, Ahlm C, et al. Cerebrospinal fluid biomarkers of brain injury, inflammation and synaptic autoimmunity predict long-term neurocognitive outcome in herpes simplex encephalitis. Clin Microbiol Infect. 2021;27(8):1131–1136. doi: 10.1016/j.cmi.2020.09.031. 32979577

[13] Grahn A, Hagberg L, Nilsson S, et al. Cerebrospinal fluid biomarkers in patients with varicella-zoster virus CNS infections. J Neurol. 2013;260(7):1813–1821. doi: 10.1007/s00415-013-6883-5. 23471614

[14] Hampel H, Blennow K, Shaw LM, et al. Total and phosphorylated tau protein as biological markers of alzheimer’s disease. Exp Gerontol. 2010;45(1):30–40. doi: 10.1016/j.exger.2009.10.010. 19853650 PMC2815003

[15] Sulik A, Toczylowski K, Kulczynska-Przybik A, et al. Amyloid and tau protein concentrations in children with meningitis and encephalitis. Viruses. 2022;14(4):725. doi: 10.3390/v14040725. 35458457 PMC9027807

[16] Toczylowski K, Wojtkowska M, Sulik A. Enteroviral meningitis reduces CSF concentration of Aβ42, but does not affect markers of parenchymal damage. Eur J Clin Microbiol Infect Dis. 2019;38(8):1443–1447. doi: 10.1007/s10096-019-03569-0. 31093802 PMC6647500

[17] Brittain-Long R, Nord S, Olofsson S, et al. Multiplex real-time PCR for detection of respiratory tract infections. J Clin Virol. 2008;41(1):53–56. doi: 10.1016/j.jcv.2007.10.029. 18093871 PMC7172039

[18] Lindström J, Elfving K, Lindh M, et al. Assessment of the FilmArray ME panel in 4199 consecutively tested cerebrospinal fluid samples. Clin Microbiol Infect. 2022;28(1):79–84. doi: 10.1016/j.cmi.2021.05.017. 34015534

[19] Gobom J, Parnetti L, Rosa-Neto P, et al. Validation of the LUMIPULSE automated immunoassay for the measurement of core AD biomarkers in cerebrospinal fluid. Clin Chem Lab Med. 2022;60(2):207–219. doi: 10.1515/cclm-2021-0651. 34773730

[20] Rosengren LE, Ahlsén G, Belfrage M, et al. A sensitive ELISA for glial fibrillary acidic protein: application in CSF of children. J Neurosci Methods. 1992;44(2-3):113–119. doi: 10.1016/0165-0270(92)90004-w. 1474847

[21] Ayukekbong J, Kabayiza J-C, Lindh M, et al. Shift of enterovirus species among children in Cameroon–identification of a new enterovirus, EV-A119. J Clin Virol. 2013;58(1):227–232. doi: 10.1016/j.jcv.2013.07.005. 23895932

[22] Larsson SB, Vracar D, Karlsson M, et al. Epidemiology and clinical manifestations of different enterovirus and rhinovirus types show that EV-D68 may still have an impact on severity of respiratory infections. J Med Virol. 2022;94(8):3829–3839. doi: 10.1002/jmv.27767. 35403229 PMC9321759

[23] Kumar S, Stecher G, Li M, et al. MEGA X: molecular evolutionary genetics analysis across computing platforms. Mol Biol Evol. 2018;35(6):1547–1549. doi: 10.1093/molbev/msy096. 29722887 PMC5967553

[24] Krut JJ, Zetterberg H, Blennow K, et al. Cerebrospinal fluid alzheimer’s biomarker profiles in CNS infections. J Neurol. 2013;260(2):620–626. doi: 10.1007/s00415-012-6688-y. 23052602

[25] Yamada K, Holth JK, Liao F, et al. Neuronal activity regulates extracellular tau in vivo. J Exp Med. 2014;211(3):387–393. doi: 10.1084/jem.20131685. 24534188 PMC3949564

[26] Ratuszny D, Suhs KW, Novoselova N, et al. Identification of cerebrospinal fluid metabolites as biomarkers for enterovirus meningitis. Int J Mol Sci. 2019;20(2):337. 10.3390/ijms20020337 PMC6359617 30650575

[27] Di Stefano A, Alcantarini C, Atzori C, et al. Cerebrospinal fluid biomarkers in patients with Central nervous system infections: a retrospective study. CNS Spectr. 2020;25(3):402–408. doi: 10.1017/S1092852919000981. 31130152

[28] Sjögren M, Gisslén M, Vanmechelen E, et al. Low cerebrospinal fluid beta-amyloid 42 in patients with acute bacterial meningitis and normalization after treatment. Neurosci Lett. 2001;314(1-2):33–36. doi: 10.1016/s0304-3940(01)02285-6. 11698140

[29] Zetterberg H. Biofluid-based biomarkers for alzheimer’s disease-related pathologies: an update and synthesis of the literature. Alzheimers Dement. 2022;18(9):1687–1693. doi: 10.1002/alz.12618. 35213777 PMC9514308

[30] Gisslén M, Krut J, Andreasson U, et al. Amyloid and tau cerebrospinal fluid biomarkers in HIV infection. BMC Neurol. 2009;9(1):63. doi: 10.1186/1471-2377-9-63. 20028512 PMC2807422

[31] Dotevall L, Hagberg L, Karlsson JE, et al. Astroglial and neuronal proteins in cerebrospinal fluid as markers of CNS involvement in lyme neuroborreliosis. Eur J Neurol. 1999;6(2):169–178. doi: 10.1111/j.1468-1331.1999.tb00010.x. 10053229

[32] Studahl M, Rosengren L, Günther G, et al. Difference in pathogenesis between herpes simplex virus type 1 encephalitis and tick-borne encephalitis demonstrated by means of cerebrospinal fluid markers of glial and neuronal destruction. J Neurol. 2000;247(8):636–642. doi: 10.1007/s004150070134. 11041333

[33] Posnakoglou L, Tatsi E-B, Chatzichristou P, et al. Molecular epidemiology of enterovirus in children with Central nervous system infections. Viruses. 2021;13(1):100. doi: 10.3390/v13010100. 33450832 PMC7828273

[34] Bodilsen J, Mens H, Midgley S, et al. Enterovirus meningitis in adults: a prospective nationwide Population-Based cohort study. Neurology. 2021;97(5):e454–e63. doi: 10.1212/WNL.0000000000012294. 34088872

[35] Verboon-Maciolek MA, Krediet TG, Gerards LJ, et al. Severe neonatal parechovirus infection and similarity with enterovirus infection. Pediatr Infect Dis J. 2008;27(3):241–245. doi: 10.1097/INF.0b013e31815c1b07. 18277927

[36] Dunn JJ. Enteroviruses and parechoviruses. Microbiol Spectr. 2016;4(3). doi: 10.1128/microbiolspec.DMIH2-0006-2015. 27337462

