# Supplementary material for: Biomarkers and genotypes in patients with Central nervous system infection caused by enterovirus
Source: Infect Dis (Lond). 2024 May 17;56(9):722–31. doi: 10.1080/23744235.2024.2345712 (PMC11371261; doi:10.1080/23744235.2024.2345712)
Supplement: Supplemental Material [file INFD_A_2345712_SM4594.pdf]

Title: Biomarkers and genotypes in patients with central nervous system infection caused by enterovirus.

Journal: European Journal of Clinical Microbiology & Infectious Diseases

Authors:

Karolina Alsén[1,2], Marianela Patzi Churqui[2,12], Helene Norder[2,12], Karolina Rembeck[1,2],

Henrik Zetterberg[3,4,5,6,7,8], Kaj Blennow[3,4,9,10], Fredrika Sahlgren[11], Anna Grahn[1,2]

Affiliations:

[1] Institute of Biomedicine, Department of Infectious Diseases, University of Gothenburg, Gothenburg, Sweden

[2] Sahlgrenska University Hospital, Department of Infectious diseases , Västra Götaland Region, Gothenburg, Sweden

[3] Inst. of Neuroscience and Physiology, University of Gothenburg, Mölndal, Sweden

[4] Clinical Neurochemistry Lab, Sahlgrenska University Hospital, Mölndal, Sweden

[5] Department of Neurodegenerative Disease, UCL Institute of Neurology, Queen Square, London, UK

[6] UK Dementia Research Institute at UCL, London, UK

[7] Hong Kong Center for Neurodegenerative Diseases, Clear Water Bay, Hong Kong, China

[8] Wisconsin Alzheimer's Disease Research Center, University of Wisconsin School of Medicine and Public Health, University of Wisconsin-Madison, Madison, WI 53792, USA

[9] Institut du Cerveau et de la Moelle épinière (ICM), Pitié-Salpêtrière Hospital, Sorbonne Université, Paris, France

[10] University of Science and Technology of China, Hefei, Anhui, P.R. China

[11] Skaraborg Hospital Skövde, Sweden

[12] Sahlgrenska University Hospital, Department of Clinical Microbiology, Region Västra Götaland, Gothenburg, Sweden

Corresponding author:

Email: Karolina.alsen@vgregion.se

Address: Sahlgrenska University Hospital, Östra

Diagnosvägen 21

SE-416 85 Gothenburg Sweden

Supplementary table 2. List of primers used to amplify 5'UTR and VP1 region and their specific annealing (An) and extension (Ex) temperatures.

Primer	Sequence	Region	PCR step	An T	Ex T
**EVP4AS**	GGTAAYTTCCACCAGCANCC	5'UTR	PCR1	57°C	72°C
**EV140S**	GARCAAGYACTYCTGTYWCCCCGG	5'UTR	PCR1/semi-nested PCR	57°C	72°C
**EV170AS**	ACACGGACACCCAAAGTAGTCGGTTCC	5'UTR	semi-nested PCR	57°C	72°C
**ENT224S**	GCIATGYTIGGIACICAYRT	VP1	PCR1	42°C	60°C
**ENT222AS**	CICCIGGIGGIAYRWACAT	VP1	PCR1	42°C	60°C
**AN89S**	CCAGCACTGACAGCAGYNGARAYNGG	VP1	Nested PCR	60°C	72°C
**AN88S**	TACTGGACCACCTGGNGGNAYRWACAT	VP1	Nested PCR	60°C	72°C
