# Supplementary material for: Biomarkers and genotypes in patients with Central nervous system infection caused by enterovirus
Source: Infect Dis (Lond). 2024 May 17;56(9):722–31. doi: 10.1080/23744235.2024.2345712 (PMC11371261; doi:10.1080/23744235.2024.2345712)
Supplement: Supplemental Material [file INFD_A_2345712_SM4452.pdf]

Title: Biomarkers and genotypes in patients with central nervous system infection caused by enterovirus.

Journal: European Journal of Clinical Microbiology & Infectious Diseases

Authors:

Karolina Alsén[1,2], Marianela Patzi Churqui[2,12], Helene Norder[2,12], Karolina Rembeck[1,2],

Henrik Zetterberg[3,4,5,6,7,8], Kaj Blennow[3,4,9,10], Fredrika Sahlgren[11], Anna Grahn[1,2]

Affiliations:

[1] Institute of Biomedicine, Department of Infectious Diseases, University of Gothenburg, Gothenburg, Sweden

[2] Sahlgrenska University Hospital, Department of Infectious diseases , Västra Götaland Region, Gothenburg, Sweden

[3] Inst. of Neuroscience and Physiology, University of Gothenburg, Mölndal, Sweden

[4] Clinical Neurochemistry Lab, Sahlgrenska University Hospital, Mölndal, Sweden

[5] Department of Neurodegenerative Disease, UCL Institute of Neurology, Queen Square, London, UK

[6] UK Dementia Research Institute at UCL, London, UK

[7] Hong Kong Center for Neurodegenerative Diseases, Clear Water Bay, Hong Kong, China

[8] Wisconsin Alzheimer's Disease Research Center, University of Wisconsin School of Medicine and Public Health, University of Wisconsin-Madison, Madison, WI 53792, USA

[9] Institut du Cerveau et de la Moelle épinière (ICM), Pitié-Salpêtrière Hospital, Sorbonne Université, Paris, France

[10] University of Science and Technology of China, Hefei, Anhui, P.R. China

[11] Skaraborg Hospital Skövde, Sweden

[12] Sahlgrenska University Hospital, Department of Clinical Microbiology, Region Västra Götaland, Gothenburg, Sweden

Corresponding author:

Email: Karolina.alsen@vgregion.se

Address: Sahlgrenska University Hospital, Östra

Diagnosvägen 21

SE-416 85 Gothenburg Sweden

Supplementary table 1. Number of EV isolates three cell lines from adult patients with CNS infection during 2010-2018. NSI stands for not successfully isolated

EV type	GMK<br>N (%)	Caco-2<br>N (%)	MRC-5<br>N (%)
E30	1 (2.6)	9 (23.7)	0
CV-B5	1 (2.6)	5 (13.1)	2 (5.3)
E6	2 (5.3)	2 (5.3)	2 (5.3)
**Sub total**	**4 (10.5)**	**16 (42.1)**	**4 (10.5)**
CPE/NSI*	7 (18.5)	5 (13.1)	9 (23.7)
No PCE/NSI	27 (71)	17 (44.8)	25 (65.7)
